# Chronic activation of PPARα with fenofibrate reduces autophagic proteins in the liver of mice independent of FGF21

**DOI:** 10.1371/journal.pone.0173676

**Published:** 2017-04-19

**Authors:** Eunjung Jo, Songpei Li, Qingning Liang, Xinmei Zhang, Hao Wang, Terence P. Herbert, Trisha A. Jenkins, Aimin Xu, Ji-Ming Ye

**Affiliations:** 1 School of Health and Biomedical Sciences, RMIT University, Melbourne, Victoria, Australia; 2 State Key Laboratory of Pharmaceutical Biotechnology, Department of Medicine, Hong Kong University, Hong Kong, China; INRA, FRANCE

## Abstract

Autophagy is a catabolic mechanism to degrade cellular components to maintain cellular energy levels during starvation, a condition where PPARα may be activated. Here we report a reduced autophagic capacity in the liver following chronic activation of PPARα with fenofibrate (FB) in mice. Chronic administration of the PPARα agonist FB substantially reduced the levels of multiple autophagy proteins in the liver (Atg3, Agt4B, Atg5, Atg7 and beclin 1) which were associated with a decrease in the light chain LC3II/LC3I ratio and the accumulation of p62. This was concomitant with an increase in the expression of lipogenic proteins mSREBP1c, ACC, FAS and SCD1. These effects of FB were completely abolished in PPARα^-/-^ mice but remained intact in mice with global deletion of FGF21, a key downstream mediator for PPARα-induced effects. Further studies showed that decreased the content of autophagy proteins by FB was associated with a significant reduction in the level of FoxO1, a transcriptional regulator of autophagic proteins, which occurred independently of both mTOR and Akt. These findings suggest that chronic stimulation of PPARα may suppress the autophagy capacity in the liver as a result of reduced content of a number of autophagy-associated proteins independent of FGF21.

## Introduction

Autophagy is a process to degrade and recycle dysfunctional cellular components via the lysosome in order to maintain cellular homeostasis [1]. It is also important in maintaining energy during periods of starvation. Autophagy is regulated by the nutrient status of the cell via a number of nutrient-sensitive signalling pathways such as mammalian target of rapamycin (mTOR) and AMP-activated protein kinase (AMPK) pathways [2–4]. Forkhead box O (FoxO) family proteins also play an important role by controlling the expression of a number of autophagy related genes [3, 5, 6]. Another transcription factor that is critical for adaptive metabolism to starvation is peroxisome proliferator-activated receptor α (PPARα). Under physiological conditions, PPARα is activated by mobilised fatty acids (FAs) but can also be activated pharmacologically by fibrates, a class of lipid-lowering drugs [7].

PPARα is highly expressed the liver and when activated it up-regulates genes for FA oxidation and gluconeogenesis to provide fuels for the body [7]. As expected from its role in promoting catabolism, recent studies have shown that hepatic autophagy is activated via PPARα during fasting or after short-term treatment with PPARα agonists both *in vivo* and *in vitro* in hepatocytes [8, 9]. Interestingly, it has been suggested that an increased autophagy activity may be gradually subsided or even reduced over time under certain conditions [10]. Therefore, the first aim of the present study was to examine the expression of autophagic proteins in the liver of both wild-type (PPARα^+/+^) and PPARα^-/-^ mice after chronic administration of the PPARα activator fenofibrate (FB). As lipogenic proteins are up-regulated during PPARα activation [11] or by inhibition of autophagy [12], our second aim was to investigate the relationship of changes in autophagic proteins with the expression of lipogenic proteins.

It has been suggested that fibroblast growth factor 21 (FGF21) is an important mediator for the physiological effects initiated by PPARα activation [13–16] and this cytokine is up-regulated along with autophagy-related gene 5 (Atg5) [17]. Thus, our third aim was to determine whether FGF21 is required for PPARα to exert its effects on the expression of autophagic proteins using FGF21^-/-^ mice. Finally, we examined the key signalling pathways that have been suggested to regulate autophagy during the chronic activation of PPARα.

In this report we show that chronic activation of PPARα by FB reduces the expression of autophagic proteins in the liver in a manner that is entirely independent of FGF21. PPARα-induced suppression of autophagic proteins is possibly mediated by a decrease in FoxO1 expression rather than through changes in the activity of mTOR or Akt. These findings suggest a need to further investigate the dynamic changes of hepatic autophagy during PPARα activation and associated implications for lipid metabolism.

## Materials and methods

### Animals

The studies were conducted in male mice starting at an age of 10–12 weeks, including wild-type (PPARα^+/+^) and PPARα^-/-^ on the background of C57BL/6N, and wild-type (FGF21^+/+^) and FGF21^-/-^ mice on the background of C57BL/6J originally obtained from Jackson Laboratories (Sacramento, CA, US). The mice were housed at 23±1°C in a 12-h light/dark cycle with free access to water and standard rodent diet consisting of 70% calories as starch, 10% calories as fat and 20% calories from proteins (Specialty Feeds, Australia). After 1–2 weeks of acclimatization, mice were fed the standard diet in the absence or presence of the PPARα agonist FB for 3 weeks. FB (Sigma-Aldrich, Australia) was administered as an additive to diet at a lower dose (50 mg/kg/day) relative to our previous studies to minimize the possible influence of body weight reduction. Body weight and food intake were monitored daily. Blood samples were taken from the tail veil in week 3 after 5–7 hours of fasting and the mice were culled by cervical dislocation. Liver was removed quickly (<5 seconds), weighed on a balance and immediately freeze-clamped for storage at -80°C for subsequent analysis. All animal experiments were approved by the Animal Ethics Committee of the RMIT University or the University of Hong Kong, where animal studies were performed.

### Determination of circulating levels of glucose and FGF21

Plasma glucose levels of PPARα^-/-^ mice were determined by glucose assay according to the manufacturer’s protocol (Sigma-Aldrich, Australia). The concentrations of plasma FGF21 in both FGF21^+/+^ and FGF21^-/-^ were measured by an ELISA kit (University of Hong Kong, Hong Kong) during FB induced 24 hours of fasting according to the manufacturer’s introductions [15].

### Extraction of hepatic triglycerides contents

Liver triglycerides (TG) were extracted by the method of Folch and determined by a TG GPO-PAP kit (Roche Diagnostic, Australia) as previously described [18]. Briefly, 30–40mg of each liver sample was homogenized in 4 ml of chloroform/methanol (2:1) using a glass pestle tissue grinder. After the homogenization, the samples were rotated at room temperature overnight to ensure the complete solubilisation of the liver TG. The next day, 2 ml of 0.6% NaCl was added to each sample and followed by centrifugation to separate the aqueous from the organic phases. The lower chloroform layer contained liver TG were carefully transferred into a glass vial and dried completely under the nitrogen or air at 45°C. The extract was reconstituted in absolute ethanol for the determination TG using a POLARstar microplate reader (BMG Labtech, Germany).

### Immunoblotting analysis

Immunoblotting analysis was performed as described in our recent reports [11, 18]. Briefly, liver tissues were homogenized in ice-cold RIPA lysis buffer supplemented with protease inhibitor cocktail and phosphatase inhibitor cocktail (Sigma Aldrich Pty Ltd, Australia) and DL-dithiothreitol. Protein samples were then denatured in a SDS sample buffer. Proteins of interest were analyzed by immunoblotting using specific antibodies from Cell Signaling (USA) unless indicated otherwise. Key autophagy proteins included Atg3, Atg4B, Atg5, Atg7, nucleoporin p62 (p62), light chain 3A/B (LC3A/B) and beclin-1, phosphor- (Ser2448) and total- mTOR, phosphor- (Thr389) and total- S6K, phosphor- (Thr37/46) and total- 4EBP. For investigating the mechanism pathway of FoxO1: phospho- and total- FoxO1, acetyl (D-19) FoxO1 (Santa Cruz, USA), sirtuin-silent mating type information regulation 2 homolog (SIRT1), phosphor- (Ser473) and total- Akt, phosphor- (Ser9) and total- glycogen synthase kinase 3 β (GSK3β). Key lipogenic enzymes were examined using specific antibodies including matured form of mSREBP-1c, acetyl-CoA carboxylase (ACC), fatty acid synthase (FAS) and stearoyl-CoA desaturase (SCD1) (Santa Cruz, USA). Acyl-coenzyme A oxidase 1 (ACOX1) (Santa Cruz, USA) was determined to indicate PPARα action and peroxisomal FA oxidation. Proteins were analyzed and normalized against the housekeeper glyceraldehyde 3-phophate dehydrogenase (GAPDH) and/or α-Tubulin, or its specific total form of protein. Immunolabeled proteins were visualized using a ChemiDoc densitometer and quantified by densitometry of Image Lab software (Bio-Rad Laboratories, USA) with inclusion of representative images.

### Quantitative RT-PCR

RNA was exacted the liver tissue using TRIzol Reagent (Invitrogen, Australia) and reverse transcribed using a high capacity cDNA reverse transcription kit (Applied Biosystems, Australia) according to the manufacturer’s instructions. Primers (GeneWorks, Australia) and SYBER green supermix (Bio-Rad, USA) were used for quantitative real time PCR. The primer sequences for FoxO1 were: forward 5’-TTCAATTCGCCACAATCTGTCC-3’ and reverse 5’-GGGTGATTTTCCGCTCTTGC-3’. All reactions were performed on QIAGEN Rotor-Gene Q PCR system (Germany). 18s was used as the normalizing control gene.

### Statistical analyses

Data are presented as means ± SEM. One-way analysis of variance (ANOVA) or the t-tests were used for comparison of relevant groups as needed. When significant differences were found from an ANOVA test, the Tukey-Kramer post-hoc multiple comparisons test was applied. Differences at *p*<0.05 were considered to be statistically significant.

## Results

### Effects of FB on whole-body parameters in PPARα^-/-^ and FGF21^-/-^ mice

PPARα^-/-^ mice were approximately 16% heavier compared with PPARα^+/+^ mice at the start of the experiment (25.5 ± 0.8 vs. 21.5 ± 0.2, n = 12, *p*<0.01). As shown in Table 1, plasma levels of glucose were 40% lower in untreated PPARα^-/-^ mice (*p*<0.01 vs. untreated PPARα^+/+^). Chronic administration of FB did not influence the body weight gain or food intake in either PPARα^+/+^ or PPARα^-/-^ mice. Consistent with our previous reports [11, 18], PPARα^+/+^ mice treated with FB displayed a 70% increase in liver weight (*p*<0.01 vs. untreated PPARα^+/+^ mice) but this effect were not detected in PPARα^-/-^ mice. In comparison, FGF21^-/-^ mice were 8% lighter compared with the age-matched FGF21^+/+^ mice at the start of the experiment (21.5 ± 0.2 vs. 25.5 ± 0.8, n = 12, *p*<0.01, Table 2). In both of the FGF21^+/+^ and FGF21^-/-^ mice, chronic administration of FB increased liver weight by ~80% (*p*<0.01 vs. corresponding untreated mice) but had no effect on body weight gain, food intake or plasma glucose.

**Table 1:** Metabolic responses to chronic activation of PPARα with FB in PPARα^-/-^ mice.

	PPARα^+/+^	PPARα^+/+^-FB	PPARα^-/-^	PPARα^-/-^-FB
Body weight (g) Basal After Body weight gain (g)	21.5 ± 0.1 24.2 ± 0.3 2.7 ± 0.2	21.5 ± 0.2 24.4 ± 0.4 2.9 ± 0.2	26.4 ± 0.9^††^ 28.7 ± 1.1^††^ 2.3 ± 0.2	24.5 ± 1.1 26.3 ± 1.8 1.8 ± 0.7
Food intake (g/day/mouse) Basal During treatment	3.2 ± 0.1 3.3 ± 0.2	3.2 ± 0.1 3.2 ± 0.1	4.1 ± 0.2 4.8 ± 0.2	5.0 ± 0.2 5.3 ± 0.2
Liver weight (g)	0.9 ± 0.0	1.6 ± 0.1**	1.1 ± 0.0	1.2 ± 0.1
Liver weight/Body weight (%)	4.0 ± 0.0	7.0 ± 0.0**	4.0 ± 0.0	5.0 ± 0.0
Plasma glucose level	10.0 ± 0.4	9.3 ± 0.3	6.9 ± 0.2^††^	6.0 ± 0.2^††^

PPARα^+/+^ and PPARα^-/-^ mice were administered with fenofibrate (FB, 50 mg/kg/day in diet) for 3 weeks. Data are means ± SEM (n = 5–6 mice/group).

* *p*<0.05,

** *p*<0.01 vs. vehicle control;

^††^ *p*<0.01 vs. corresponding wild-type.

**Table 2:** Metabolic responses to chronic activation of PPARα with FB in FGF21^-/-^ mice.

	FGF21^+/+^	FGF21^+/+^-FB	FGF21^-/-^	FGF21^-/-^-FB
Body weight (g) Basal After Body weight gain (g)	25.9 ± 1.4 29.5 ± 1.3 3.6 ± 0.1	25.1 ± 0.5 29.5 ± 0.6 4.4 ± 0.1	21.1 ± 1.1^†^ 24.3 ± 1.2^†^ 3.2 ± 0.1	22.0 ± 1.0^†^ 25.2 ± 1.0^†^ 3.2 ± 0.0
Food intake (g/day/mouse) Basal During treatment	4.2 ± 0.2 4.4 ± 0.2	4.1 ± 0.1 4.2 ± 0.1	3.8 ± 0.2 3.9 ± 0.1	4.1 ± 0.2 4.0 ± 0.2
Liver weight (g)	1.2 ± 0.0	2.1 ± 0.2**	1.1 ± 0.1	2.0 ± 0.1*
Liver weight/Body weight (%)	4.0 ± 0.3	7.2 ± 0.7*	4.4 ± 0.2	8.0 ± 0.2**
Blood glucose level	12.2 ± 1.6	10.5 ± 1.2	12.6 ± 1.8	13.2 ± 1.7^††^

FGF21^+/+^ and FGF21^-/-^ mice were administered with fenofibrate (FB, 50 mg/kg/day in diet) for 3 weeks. Data are means ± SEM (n = 5–6 mice/group).

* *p*<0.05,

** *p*<0.01 vs. vehicle control;

^†^ *p*<0.05,

^††^ *p*<0.01 vs. corresponding wild-type.

### Effects of FB on hepatic lipid metabolism and autophagic proteins in PPARα^+/+^ and PPARα^-/-^ mice

**Fig 1:**
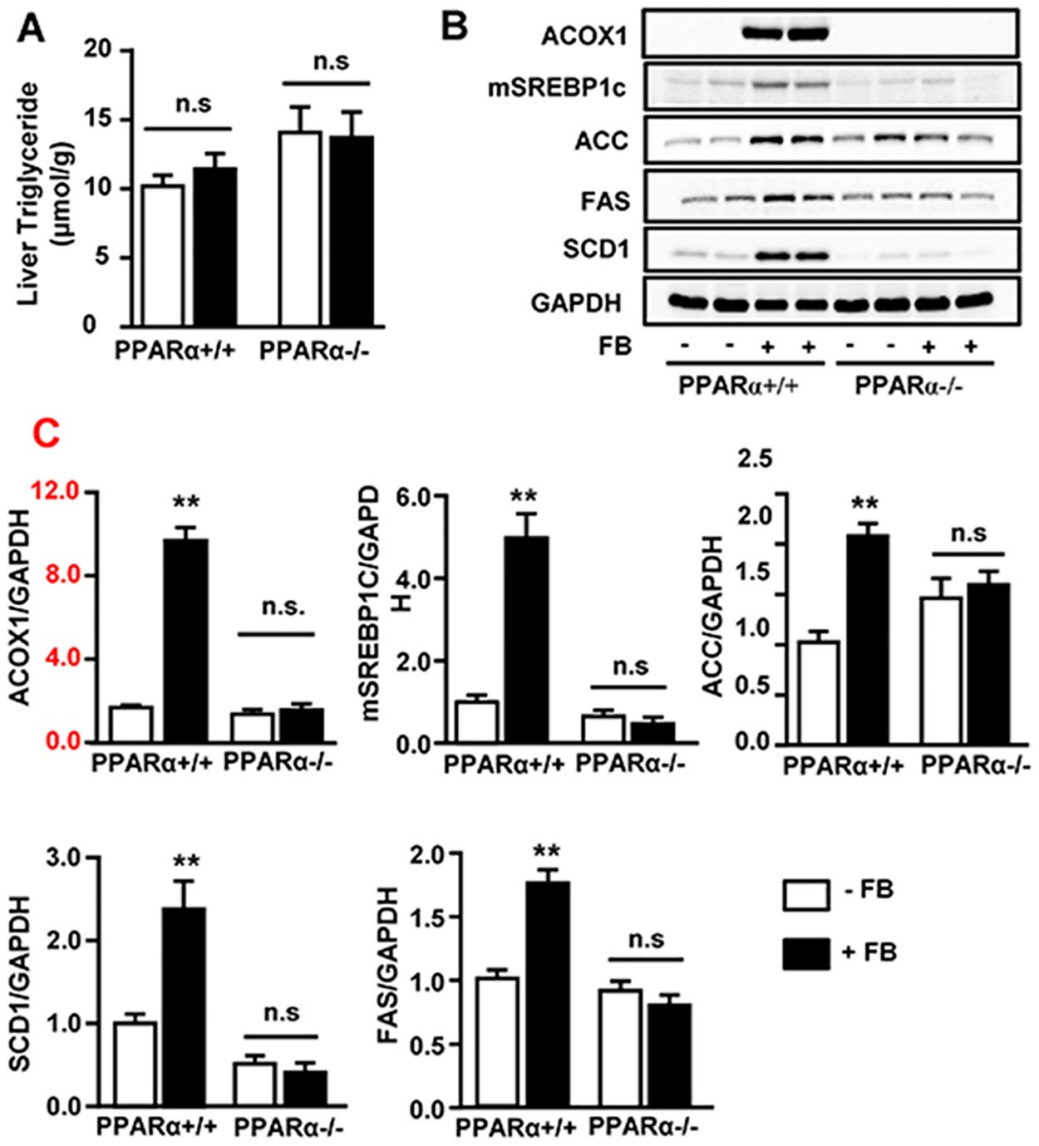
Effects of FB on hepatic TG content and the lipogenic pathway in PPARα^+/+^ and PPARα^-/-^ mice. Fenofibrate (+ FB) was administered to PPARα^+/+^ and PPARα^-/-^ mice on the background of C57BL/6N at a dose (50 mg/kg/day) for 3 weeks. **(A)** Effects on triglyceride (TG) content. **(B)** Representative images and **(C)** Quantification of Western blots for ACOX1 (peroxisomal acyl-CoA oxidase 1, PPARα activation marker) and lipogenic proteins: mSREBP1c (matured form of sterol regulatory element-binding protein 1 c), ACC (acetyl-CoA carboxylase), FAS (fatty acid synthase) and SCD1 (stearoyl-CoA desaturase 1). ** *p*<0.01 vs. control (-FB); n.s. not statistically significant. n = 5–6/group.

Chronic administration of FB had no significant effect on hepatic triglyceride (TG) content in either PPARα^+/+^ or PPARα^-/-^ mice (Fig 1A). In PPARα^+/+^ mice, FB up-regulated the protein expression of ACOX1 (~2.2-fold, Fig 1B and 1C), a PPARα responsive enzyme catalysing FA oxidation in peroxisomes. The levels of lipogenic proteins mature SREPB-1c, ACC, FAS and SCD1 were increased by 2–3 fold (*p*<0.01). However, these increases were abolished in PPARα^-/-^ mice, confirming the requirement of PPARα for FB to up-regulate the lipogenic pathway.

**Fig 2:**
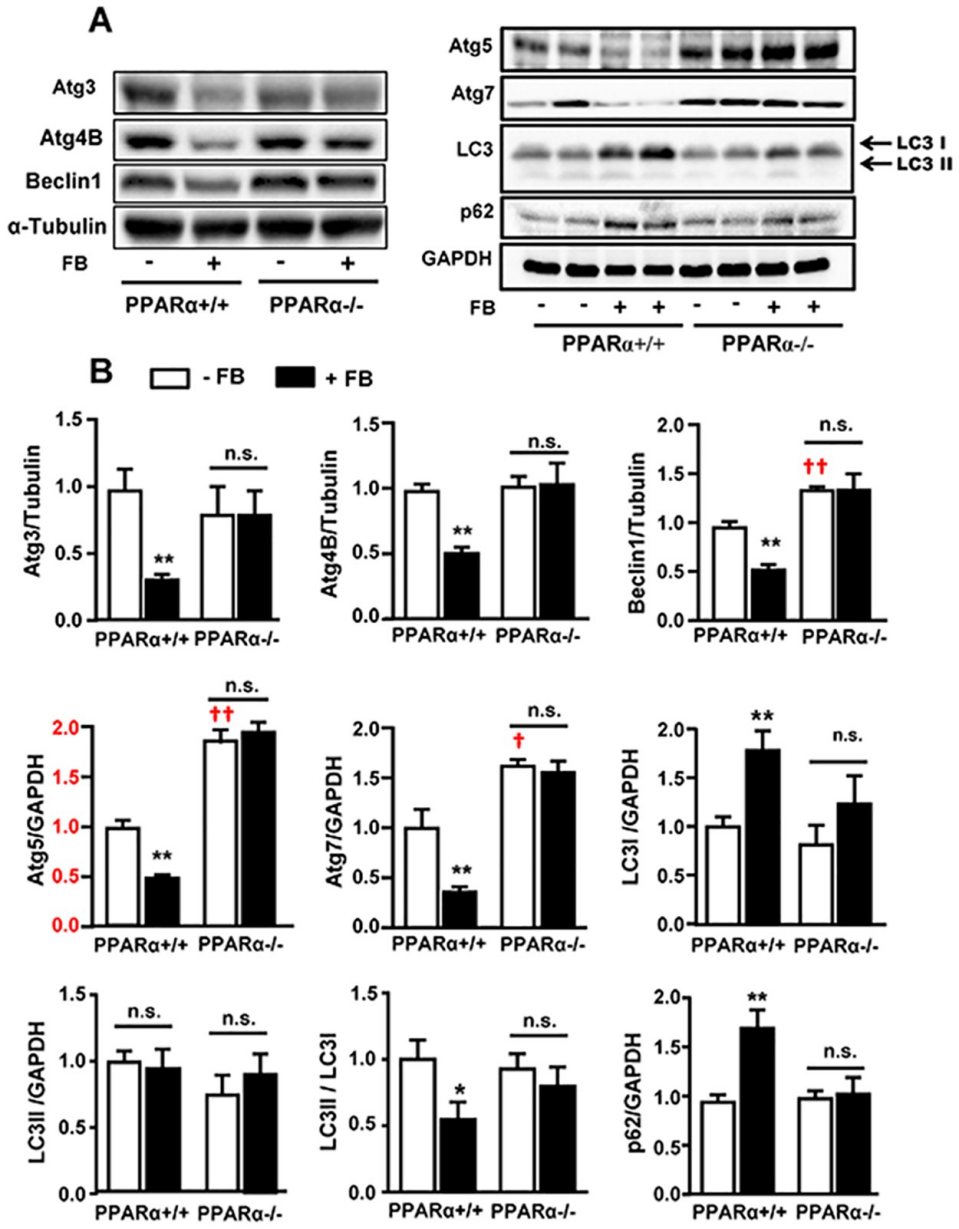
Effects of FB on hepatic autophagic proteins in PPARα^+/+^ and PARα^-/-^ mice. Experiments were conducted as described in Fig 1. **(A)** Representative of images and **(B)** quantification of the Western blots for key autophagic proteins. Atg3 (autophagy-related gene protein 3), Atg4B (autophagy-related gene protein 4B), Atg5 (autophagy-related gene protein 5), Atg7 (autophagy-related gene protein 7), LC3 (microtubule-associated protein light chain 3) and p62 (polyubiquitin-binding protein p62). * *p*<0.05, ** *p*<0.01 vs control (-FB); † *p*<0.05, †† *p*<0.01 vs untreated PPARα^+/+^ mice; n.s. not statistically significant. n = 5–6/group.

In PPARα^+/+^ mice, chronic administration of FB reduced the protein levels of Atg3, Atg4B, Atg5, Atg7 and beclin1 by more than 50% (all *p*<0.01, Fig 2), suggesting a decreased autophagy capacity. The LC3II to LC3I ratio (indicative of autophagosome formation) was inhibited by ~50% (due to the accumulation of LC3I) whereas p62 protein (indicator of non-degraded aggregates) was accumulated by ~70%. In the liver of PPARα^-/-^ mice, there were increases in the expression of beclin 1 (~40%), Agt5 (~80%) and Agt7 (~60%) compared to untreated PPARα^+/+^ mice (all p<0.05) but other measured autophagic proteins were similar. However, chronic administration of FB in PPARα^-/-^ mice had no effects on the level of autophagic proteins, indicating the requirement of PPARα activation for FB to mediate the effect of FB to reduce the level of autophagic proteins.

### Effects of FB on hepatic lipid metabolism and autophagic proteins in FGF21^+/+^ and FGF21^-/-^ mice

**Fig 3:**
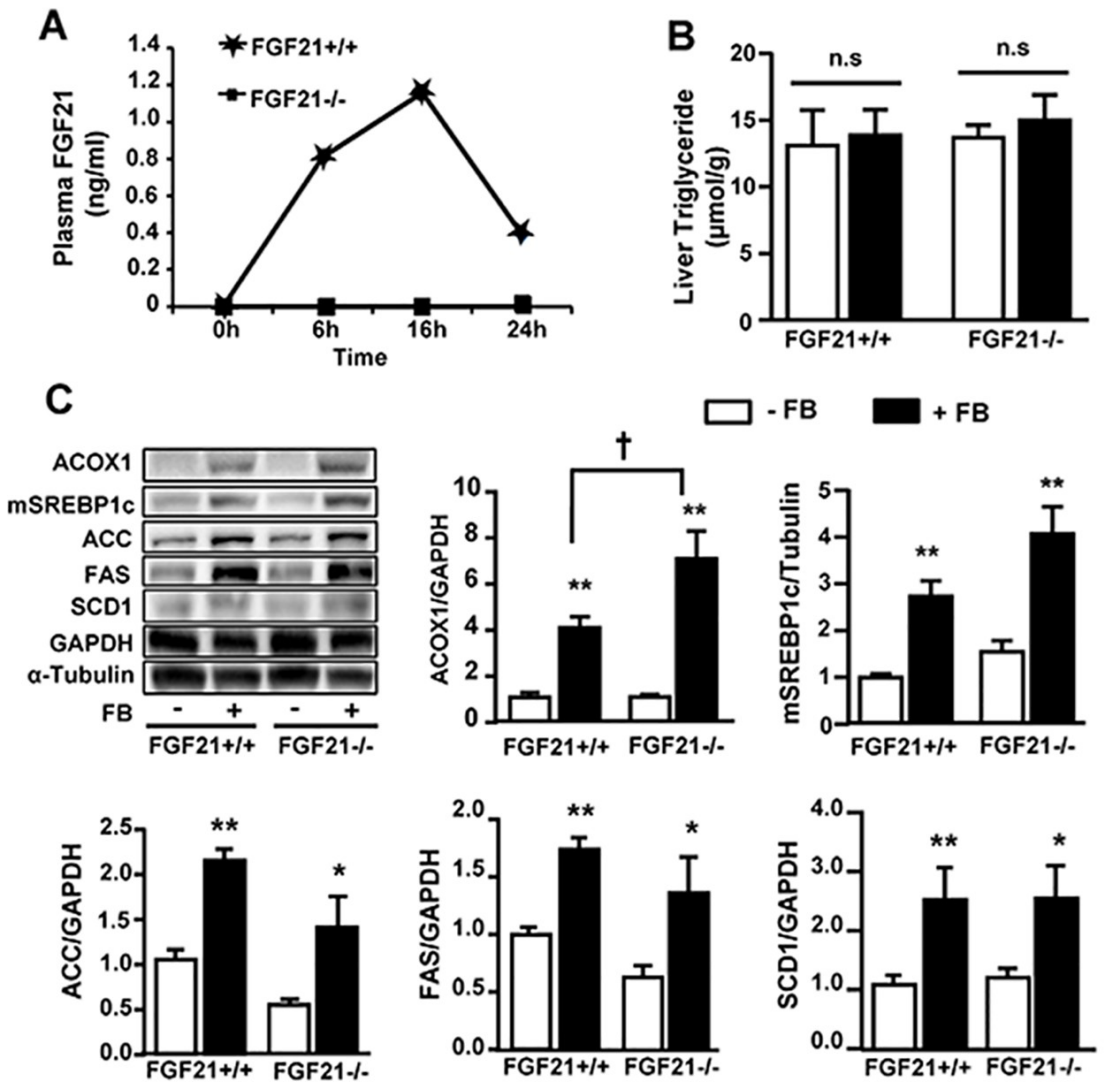
Effects of FB on hepatic TG content and lipogenic proteins in FGF21^+/+^ and FGF21^-/-^ mice. **(A)** Plasma levels of FGF21 in response to fasting. For **B** and **C**, FB was administered to FGF21^+/+^ and FGF21^-/-^ mice on the background of C57BL/6J at a dose (50 mg/kg/day) for 3 weeks. **(B)** Liver TG content. **(C)** Representative images and quantification of the Western blots for ACOX1, mSREBP1c, ACC, FAS and SCD1. * *p*<0.05, ** *p*<0.01 vs control (-FB); n.s. not statistically significant, n = 5–6/group.

**Fig 4:**
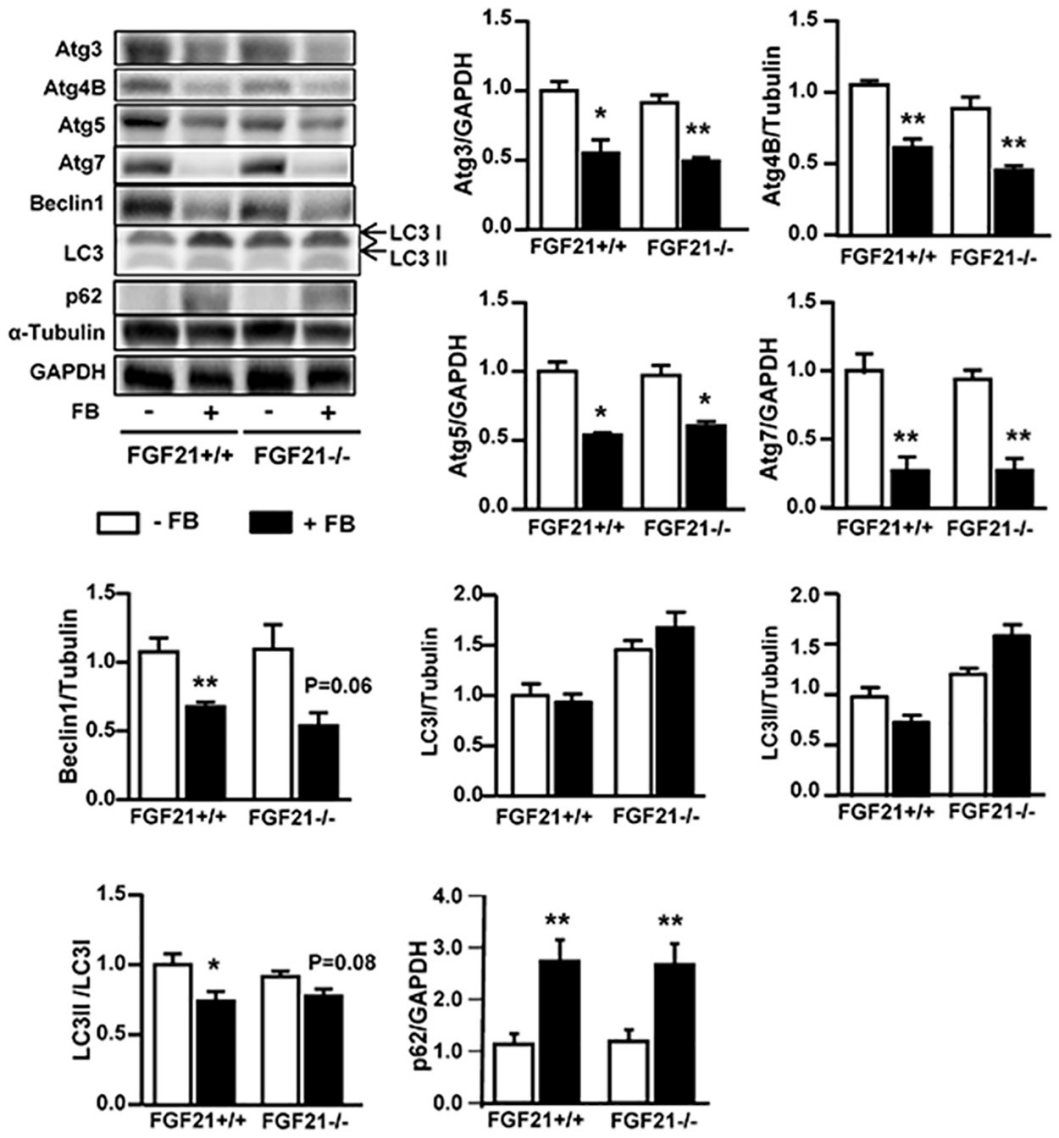
Effects of FB on the content of autophagic proteins in the liver of FGF21^+/+^ and FGF21^-/-^ mice. FGF21^+/+^ and FGF21^-/-^ mice were administrated with FB for 3 weeks as described in the methods. The liver samples were collected for Western blots for autophagic proteins including Atg3, Atg4B, Atg5, Atg7, Beclin-1, LC3 and p62. * *p*<0.05; ** *p*<0.01 vs group (- FB), n = 5–6/group.

We next investigated whether or not FGF21 may be required for the effects of FB on those lipogenic and autophagic proteins. In FGF21^-/-^ mice, fasting-induced increase in plasma levels of FGF21 was completely diminished (Fig 3A), confirming the lack of FGF21 after its deletion. While liver TG content was not affected (Fig 3B) after chronic administration of FB, the effect of FB in increasing ACOX1 protein was significantly enhanced in FGF21^-/-^ mice (*p*<0.05 vs. FB-treated FGF21^+/+^ mice, Fig 3C). Despite this, the effects of FB to up-regulate mSREPB-1c, ACC, FAS and SCD1 were similar in FGF21^-/-^ mice compared to FGF21^+/+^ mice. Atg3, Atg4B, Atg5, Atg7 and beclin1 were reduced (by more than 50%) along with an increase in p62 (2-fold) to the similar extent in both FGF21^+/+^ and FGF21^-/-^ mice after chronic administration of FB (Fig 4). These results suggest that FGF21 is not required for PPARα-induced expression of proteins in the lipogenic and autophagic pathways in the liver.

### Effects of FB on the mTOR and insulin signalling pathways in PPARα^+/+^ and PPARα^-/-^ mice

**Fig 5:**
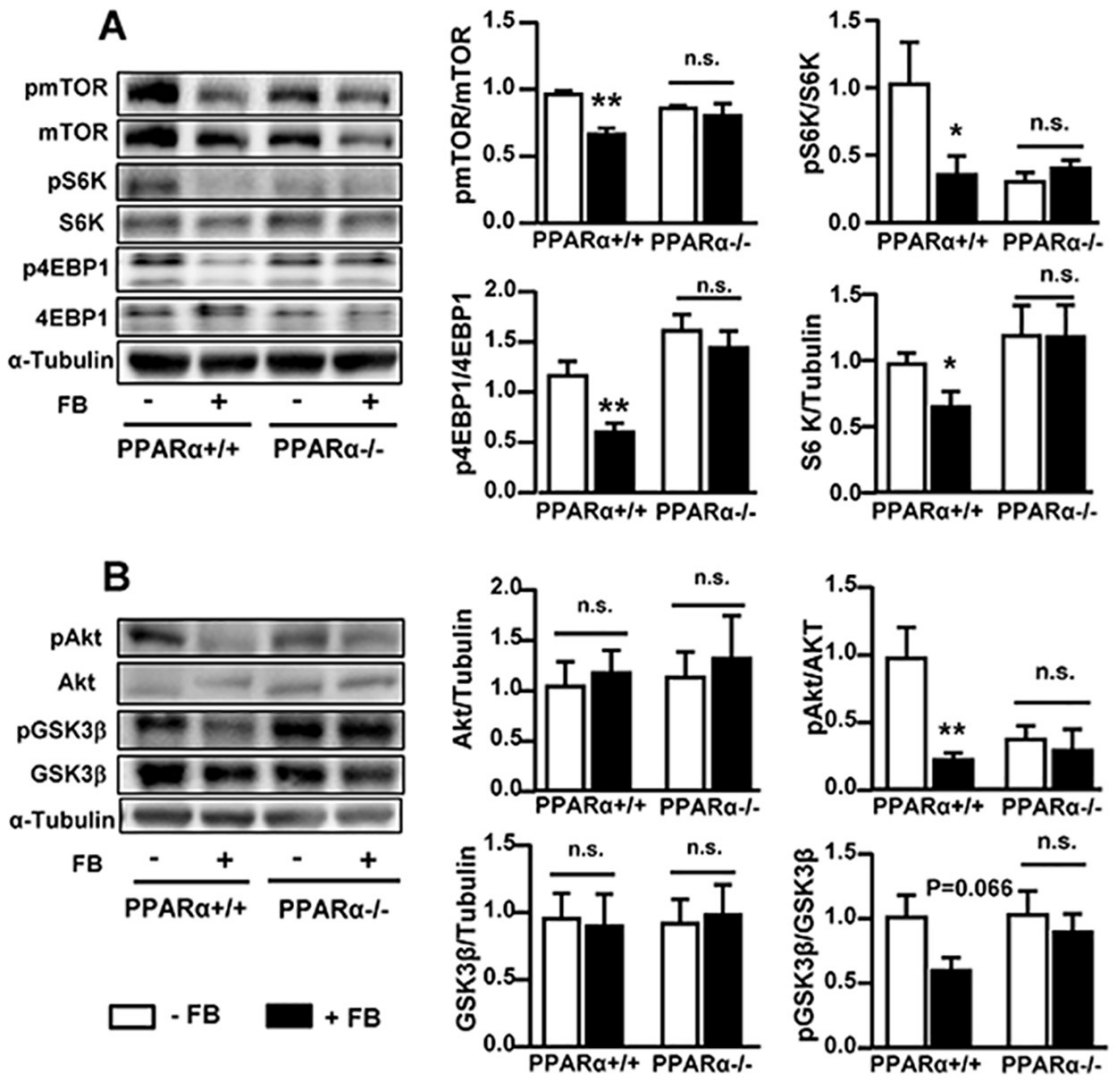
Effects of FB on the autophagy upstream pathways in PPARα^+/+^ and PARα^-/-^ mice. Liver samples were collected after 3 weeks of FB administration for Western blotting. **(A)** Effects of FB on key proteins of the mTOR pathway: mTOR (mammalian target of rapamycin), S6K (P70S6 serine/threonine kinase), 4EBP1 (eukaryotic translation initiation factor 4E-biniding protein). **(B)** Effects of FB on key proteins the insulin signaling pathway: Akt (protein kinase B) and GSK3β (glycogen synthase kinase 3 β). * *p*<0.05, ** *p*<0.01 control (-FB); n.s. not statistically significant, n = 5–6/group.

We next examined the effects of FB on mTOR and insulin signalling in both PPARα^+/+^ and PPARα^-/-^ mice as the activation of these pathways can inhibit autophagy activity. In PPARα^+/+^ mice, FB inhibited the phosphorylation of mTOR (by ~20%) and its downstream effectors S6K (by ~65%) and 4EBP1 (~50%) (all *p*<0.05) (Fig 5). The phosphorylation of Akt was significantly inhibited with a similar trend of change for GSK3β. However, these effects by FB were all abolished in PPARα^-/-^ mice. These results suggest that the changes in the expression of autophagic proteins by PPARα activation with FB are not due to the activation of the mTOR or insulin signalling pathway.

### Effects of FB on FoxO1, SIRT1 and HDAC4 in PPARα^+/+^ and PPARα^-/-^ mice

**Fig 6:**
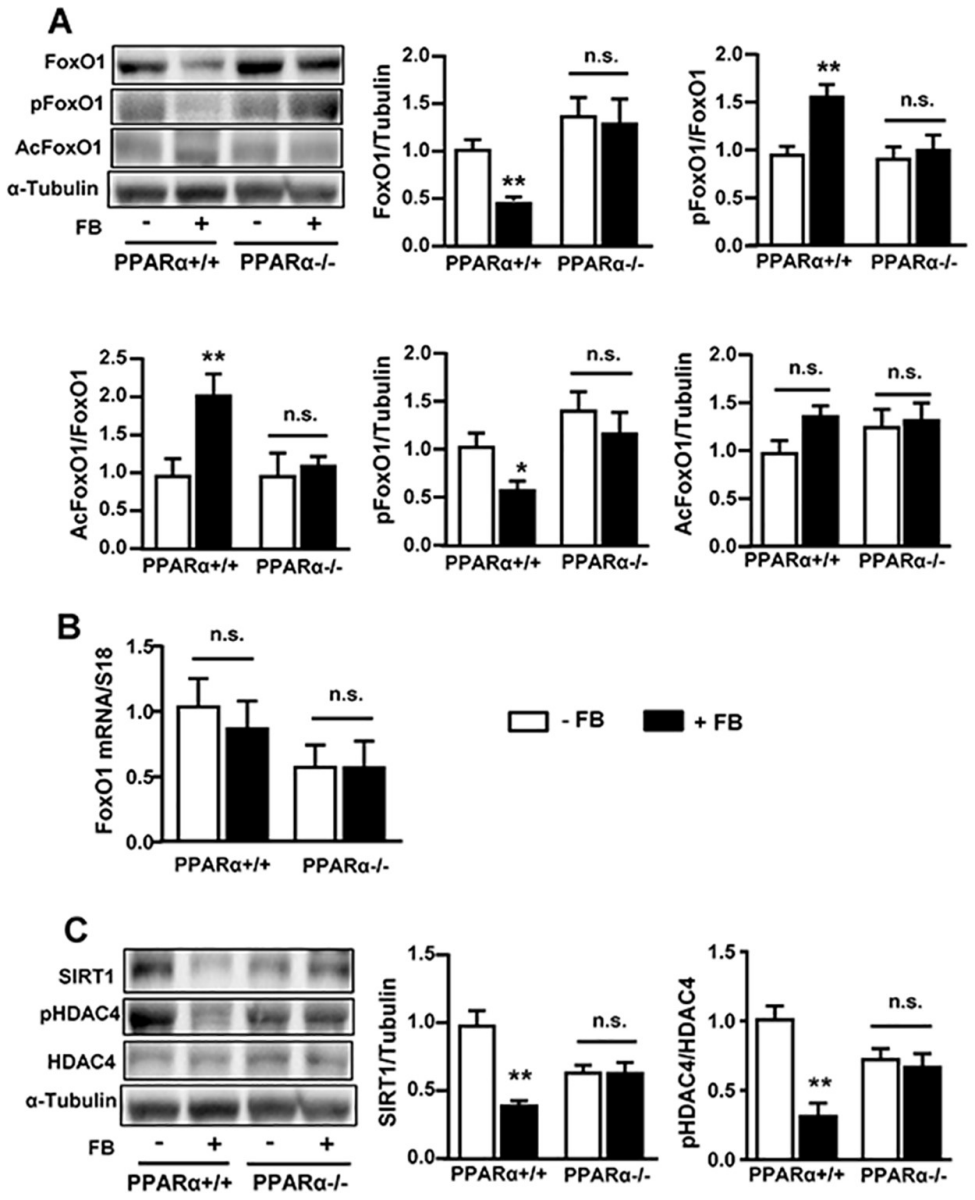
Effects of FB on FoxO1 and SIRT1 in PPARα^+/+^ and PARα^-/-^ mice. Liver samples were collected after 3 weeks of FB administration. **(A)** Effects on the content, phosphorylation and acetylation (Ac) of FoxO1 the protein level. **(B)** Effects on the level of mRNA of FoxO1. **(C)** Effects on deacetylases, SIRT1 (silent mating type information regulation 2 homolog or NAD-dependent deacetylase sirtuin-1) and HDAC4 (histone deacetylases 4). * *p*<0.05, ** *p*<0.01 vs control (-FB); n.s. not statistically significant; n = 5–6/group.

We next investigated whether the reduced autophagic proteins after chronic administration of FB may be mediated by FoxO1, a critical transcription factor regulating autophagic proteins. In PPARα^+/+^ mice, the expression of FoxO1 was markedly reduced (by ~60%) by the activation of PPARα with FB (*p*<0.01, Fig 6A). This reduction was associated with increases in the ratios of both p-FoxO1/FoxO1 (~1.5 fold) and Ac-FoxO1/Foxo1 (~2 fold). However, the expression of FoxO1 mRNA of was not altered by FB treatment (Fig 6B). Concomitant with the increased acetylation of FoxO1, the level of SIRT1 (a Class III deacetylase) was reduced (~60%) and the phosphorylation of histone deacetylase 4 (HDAC4, a Class I deacetylase) was decreased (~70%) (Fig 6C). In PPARα^-/-^ mice, all of these effects of FB were abolished, indicating that these observed changes were entirely dependent upon PPARα.

## Discussion

The present study investigated the effect of chronic activation of PPARα on liver autophagy in mice and the results revealed several novel findings. Firstly, chronic administration of FB PPARα-dependently down-regulate multiple autophagic proteins while up-regulating lipogenic proteins. Secondly, these PPARα-dependent effects were entirely independent of FGF21, a key downstream mediator of PPARα. Thirdly, PPARα-induced the reductions in autophagic proteins was associated with a reduction in FoxO1 (master transcriptional factor for autophagic proteins). These findings suggest that chronic activation of PPARα may reduce the capacity for autophagy in the liver by reducing autophagic proteins via FoxO1 to influence hepatic lipid metabolism.

PPARα plays an important role in regulating a wide range of metabolic effects in the liver. Under physiological conditions, it is activated by elevated FAs to promote FA oxidation by up-regulating enzymes in mitochondria and peroxisomes including ACOX1 [11, 19]. It has been recently reported that short-term (24–48 hrs) activation of PPARα in primary hepatocytes increase autophagic activity [9]. However, the effect of long-term activation of PPARα on autophagy is not clear. The present study revealed that chronic administration of the PPARα agonist FB down-regulates the expression of multiple autophagic proteins (i.e. Atg3, Atg4B, Atg5, Atg7 and beclin1). Along with this, autophagic activity may be compromised as suggested by the reduced LC3II/LC3I ratio and accumulation of p62, a polyubiquitin-binding protein p62 (SQSTM1) degraded by autophagy [1, 20]. These effects are specific to activation of PPARα (indicated by increased ACOX1) because they are completely abolished in PPARα^-/-^ mice. These results lead us to suggest that persistent stimulation of PPARα may result in a negative feedback mechanism to down-regulate autophagic proteins in the liver. Consistent with this notion, some autophagic proteins (beclin 1, Agt5 and Agt7) were found to be increased in PPARα^-/-^ in the absence of FB, suggesting a regulation of PPARα on the expression of autophagic proteins under physiological conditions.

Our previous work showed that chronic administration of FB promotes *de novo* lipogenesis in the liver by up-regulating lipogenic proteins in mice in the presence of PPARα [18]. The results from the PPARα^-/-^ mice in the present study confirmed that the up-regulation of lipogenic proteins in response to FB is mediated specifically by PPARα. It has been shown that an inhibition of autophagy can lead to increased *de novo* lipogenesis such as in high fructose feeding [12]. Similarly, there is an inverse relationship of autophagic activity with *de novo* lipogenesis in response to chronic activation of PPARα. The results from this study suggest that the altered autophagy by chronic activation of PPARα may also impact on *de novo* lipogenesis in the liver.

PPARα is known to play a critical role in promoting hepatic gluconeogenesis and FA oxidation mediated by FGF21 during starvation [15, 21] where autophagy activity is also altered. However, the observed changes in autophagic proteins (Atg3, Atg4B, Atg5, Atg7, beclin1, LC3 and p62) and lipogenic proteins (mSREBP1, ACC, FAS and SCD1) by chronic activation PPARα with FB remained intact in FGF21^-/-^ mice. These findings indicate that PPARα-induced expressions of autophagic and lipogenic proteins are independent of its downstream mediator FGF21.

Autophagy activity can be regulated by the mTOR pathway [2–4]. However, chronic activation of PPARα with FB actually inhibits the mTOR pathway (indicated by reduced pmTOR, pS6K and p4EBP1). Because mTOR can be activated by the stimulation of insulin signalling [22, 23], we examined insulin signalling and found that the activity of pAkt and pGSK3β were decreased by chronic stimulation of PPARα similarly to our recent reports [11, 18]. These results together indicate that PPARα-mediated down-regulation of autophagic proteins cannot be attributed to the mTOR pathway. Such interpretation agrees with the notion that the constitutive activity of autophagy is insensitive to the mTOR pathway [10].

FoxO1 is a master transcription factor controlling the expression of autophagy proteins [3, 5, 6]. Interestingly our results showed the level of FoxO1 was markedly reduced following chronic activation of PPARα as previously reported [24], suggesting that the down-regulation of autophagic proteins is possibly due to the inhibition of FoxO1. FoxO1 is degraded once translocated to the cytosol from the nucleus once phosphorylation and acetylation [5, 6]. Indeed, the reduction in FoxO1 protein level was associated with increases in its phosphorylation and acetylation but there was no change in the mRNA expression of FoxO1. Thus, we speculate that the reduced FoxO1 content is due to an increased degradation promoted by its phosphorylation and/or acetylation. It has been suggested that FoxO3 may also induce autophagy by controlling the transcription of LC3 and Bnip3 [25, 26]. Additional studies are warranted to investigate the role of FoxO3 in PPARα-induced changes in the expression of autophagic proteins in the liver. However, we do not rule out the direct effect on autophagy proteins (including the degradation of autophagic proteins) from PPARα because the regulation of autophagy genes is multifactorial. The present study focused on the chronic effect of PPARα activation (3 weeks) as opposed to acute effect reported by Lee et al [9] and Jiao et al. [8]. The different time points to capture the dynamic changes in autophagy during PPARα activation may also contribute to the discrepancy.

Several recent studies suggest that an increase in the acetylation of FoxO1 may result from the inhibition of deacetylases [27, 28]. Our recent work showed that the acetylation of FoxO1 can be increased as a result of the suppression of the deacetylases SIRT1 and HDAC4 [29]. In the present study, level of SIRT1 and HDAC4 phosphorylation were reduced chronic activation of PPARα. As deacetylase activity is a mechanism to retain FoxO1 in the nucleus of hepatocytes [30], our findings from this study suggest that the chronic activation of PPARα may promote the acetylation FoxO1 via suppressing of SIRT1 and HDAC4 as a mechanism contributing to the degradation of FoxO1.

In summary, the present study suggests that chronic activation of PPARα decreases the autophagic capacity in the liver by reducing multiple autophagic proteins while increasing lipogenic proteins. These changes are independent of the PPARα downstream mediator FGF21 and the mTOR pathway. The reduction in autophagic proteins may be due to a down-regulation due to reduction in FoxO1. The suppression of SIRT1 and HDAC4 by chronic activation of PPARα may contribute to the acetylation of FoxO1. These findings may help explain why chronic activation of PPARα fails to reduce hepatic steatosis in high fat fed mice despite increased FA oxidation [11, 31]. As the fibrate class drugs are chronically used in clinics, the observations from this study suggest a need to consider their potentially impact on level of autophagic proteins.

## Supporting information

S1 File Data file. (XLSM)
